# Clinical and FDG-PET/CT Suspicion of Malignant Disease: Is Biopsy Confirmation Still Necessary?

**DOI:** 10.3390/diagnostics11030559

**Published:** 2021-03-20

**Authors:** Talitha Bent, Derya Yakar, Thomas C. Kwee

**Affiliations:** Medical Imaging Center, Departments of Radiology, Nuclear Medicine and Molecular Imaging, University of Groningen, University Medical Center Groningen, 9713 Groningen, The Netherlands; talithabent@gmail.com (T.B.); thomaskwee@gmail.com (T.C.K.)

**Keywords:** ^18^F-FDG, biopsy, cancer, malignancy, PET-CT, tumors

## Abstract

Background: Biopsy of ^18^F-fluoro-2-deoxy-D-glucose (FDG)-avid lesions suspected for malignancy remains an invasive procedure associated with a variety of risks. It is still unclear if the positive predictive value (PPV) of positron emission tomography (PET)/computed tomography (CT) is sufficiently high to avoid tissue sampling. Therefore, the purpose of this study was to determine the PPV of ^18^F-FDG-PET/CT for malignancy in patients with a clinical suspicion of active malignant disease. Methods: This single-center retrospective study included 83 patients who had undergone FDG-PET/CT within 60 days before CT- or ultrasonography-guided tissue sampling and whose request form for CT- or US-guided tissue sampling requested mutation analyses. The latter implies a high clinical suspicion of active malignant disease. The nature of each biopsied lesion was determined based on the results of the pathological analysis and/or clinical and imaging follow-up of at least 12 months. Results: In total, eighty-eight FDG-avid lesions were biopsied. The PPV of FDG-PET/CT for malignancy was 98.9% (95% CI: 93.8–99.8%). For patients with an oncological history, the PPV was 98.7% (95% CI: 92.9–99.8%), and for patients with no oncological history, the PPV was 100% (95% CI: 74.1–100.0%). There was no significant difference between the PPV of the group with and without an oncological history (*p* = 0.71). In two cases, an unsuspected malignancy was diagnosed. Conclusion: Although the PPV of FDG-PET/CT for malignancy in patients with a clinical suspicion of active malignant disease is high, biopsy remains recommended to avoid inappropriate patient management due the non-negligible chance of dealing with FDG-avid benign disease or unexpected malignancies.

## 1. Introduction

During the last three decades, the role of positron emission tomography (PET) in diagnosis, staging, and follow-up of cancer patients has been revolutionizing [1,2]. Its clinical use has particularly accelerated due to the introduction of hybrid systems, which combine PET with computed tomography (CT) [3]. The most widely used radiotracer for PET is ^18^F-fluoro-2-deoxy-D-glucose (FDG), which is taken up by viable cells in the same way as D-glucose [3,4]. However, in contrast to D-glucose, once taken up by the cell, FDG cannot enter the glycolytic pathway and accumulates intracellularly, a phenomenon called metabolic trapping [5]. Metabolic trapping in combination with increased glucose demands results in higher levels of FDG in malignant cells than in normal tissues, which can be visualized with PET [5]. Nonetheless, it should be noted that FDG is not a specific cancer tracer, since it can accumulate in benign lesions, such as inflammatory, posttraumatic, and several benign neoplastic conditions [6,7,8].

Two previous studies reported overall positive predictive values (PPVs) of FDG-PET/CT for malignancy of 72% [9] and 82.4% [10]. These numbers suggest that tissue sampling is necessary to confirm malignant disease and that no patient management decisions should be made without pathological confirmation. However, these two previous studies did not take into account the level of clinical suspicion of malignant disease when calculating the PPV of FDG-PET/CT for malignancy [9,10]. For example, in patients with bacteremia, an FDG-avid lesion is more likely to be of an infectious than of a malignant nature. On the other hand, in patients with a recent history of malignant disease, a new FDG-avid lesion can be regarded as suspicious of recurrent cancer. In the latter group of patients who are clinically suspected to suffer from malignant FDG-avid disease, it remains unclear if the PPV of FDG-PET/CT is sufficiently high to avoid tissue sampling. This information is important, because tissue sampling is an invasive procedure associated with a risk of complications, the degree of which depend on the body region of interest. Moreover, some lesions cannot be safely targeted, either due to their location or patient-related factors such as pain and inability to lie still [11], and management decisions have to be made without pathological examination in these patients.

The purpose of this study was therefore to determine the PPV of FDG-PET/CT for malignancy in patients with a clinical suspicion of active malignant disease.

## 2. Materials and Methods

### 2.1. Study Design and Patients

This single-center retrospective study received approval from the ethical review board of the University Medical Center Groningen (15 January 2019), and the need for written informed consent was waived (IRB Approval Number 201800105). All patients who underwent percutaneous tissue sampling under CT or ultrasonography (US) guidance at a single tertiary care center between April 2010 and May 2018 were potentially eligible for inclusion. Patients were only included if an FDG-PET/CT scan was performed within 60 days before CT- or ultrasonography (US)-guided tissue sampling and if the request form for CT- or US-guided tissue sampling included an order for mutation analyses. The latter implies that the abnormality or abnormalities found on the FDG-PET/CT scan were clinically highly suspicious of active malignant disease.

### 2.2. FDG-PET/CT Acquisition

PET/CT imaging and reconstruction were carried out in accordance with the guidelines set out by the European Association of Nuclear Medicine [12]. Patients fasted for a minimum of 6 h before intravenous administration of 3 MBq of FDG per kg of body weight, and blood glucose levels were verified to be less than 11 mmol/L. PET/CT images were acquired using a Biograph mCT 64-slice PET/CT system (Siemens Healthineers, Erlangen, Germany), from mid-thighs to cranial vertex, 60 min after FDG injection. Low-dose CT (100 kv, 30 mAs) was performed for attenuation correction and anatomic mapping.

### 2.3. FDG-PET/CT Interpretation

FDG-PET/CT images were reviewed by consensus reading of two radiologists using Syngo.via software (Siemens Healthineers, Erlangen, Germany). FDG uptake of biopsied lesions was visually graded using a four-point scale as: (1) slight uptake below mediastinal blood pool uptake; (2) uptake above mediastinal blood pool, but below or equal to liver uptake; (3) uptake slightly or moderately higher than liver uptake; or (4) uptake markedly higher than liver uptake. FDG-PET/CT readers were blinded to the results from pathological analysis and from follow-up.

### 2.4. Biopsy

All percutaneous histologic core needle biopsies or cytologic fine needle aspirations (the latter only for lymph nodes in the head-neck region) were performed by board-certified radiologists. For US guidance, two different systems (Xario XG, Toshiba, Tokyo, Japan or Acuson S2000, Siemens Healthineers, Erlangen, Germany) were used, with 8–14 MHz transducers. For CT guidance, a multislice system (Definition 64 AS, Siemens Healthineers, Erlangen, Germany) was used. Core needle sizes for tru-cut biopsies ranged between 10 and 18 gauge, depending on the preference of the radiologist who performed the procedure. The number of biopsy samples needed was also determined by the attending radiologist and depended on the macroscopic amount of tissue retrieved during biopsy.

### 2.5. Pathological Examination and Mutation Analysis

Pathological examination of retrieved biopsy tissue or cytological material was carried out by certified pathologists. In cases where malignant tissue was obtained, additional mutation analyses were done by next-generation sequencing. Pathologists were aware of the fact that the acquired tissue samples were from an FDG-avid lesion that was clinically suspicious for malignancy.

### 2.6. Reference Standard

The nature of each biopsied lesion was determined based on the results of the pathological analysis. Whenever the amount of tissue was deemed insufficient or inadequate to make a diagnosis, subsequent tissue samplings of the same lesion (if performed) or clinical and imaging follow-up of at least 12 months were used to determine the nature of each lesion (i.e., malignant or benign). If the nature of the lesion could not be established, it was excluded from the PPV analysis.

### 2.7. Statistical Analysis

Clinical patient data, as well as FDG-PET/CT and biopsy data, were descriptively analyzed. The PPV of FDG-PET/CT for malignancy in patients with a clinical suspicion of active malignant disease was calculated (considering all biopsied FDG-avid lesions), along with the 95% confidence intervals (CIs). Separate analyses were done in patients with and without an oncologic history, and PPVs between these two groups were compared using a Chi-squared test. All data were analyzed in IBM Statistical Package for the Social Sciences (SPSS) Version 23 (IBM Corp., Armonk, NY, USA).

## 3. Results

### 3.1. Patients

A total of 83 patients underwent FDG-PET/CT within 60 days before CT- or US-guided tissue sampling, and they all fulfilled the criteria for inclusion in this study. These 83 patients consisted of 39 men and 44 women, with a mean age ± SD of 61.4 ± 11.4 years (age range: 36–84 years). Seventy-two patients (86.7%) had an oncologic history (Table 1, the majority due to lung cancer), and these oncologic patients underwent FDG-PET/CT either for staging (*n* = 16), treatment response assessment (*n* = 23), suspicion of recurrent or progressive disease (*n* = 31), or for anatomical mapping of lesions for biopsy (*n* = 2). The remaining 11 patients (13.3%) had no oncologic history, and all of these patients underwent FDG-PET/CT for the evaluation of a newly discovered lesion on chest radiography (Table 1). Five patients, all with a history of lung cancer, underwent FDG-PET/CT with subsequent biopsy and a request for mutation analyses on two different occasions. Therefore, the total number of biopsied lesions amounted to 88. The mean time ± SD between FDG-PET/CT and biopsy was 15.2 ± 4.3 days (range: 9–21 days).

### 3.2. FDG-PET/CT

In all 88 cases in which a mutation analysis was requested, PET/CT showed multiple FDG-avid lesions in the same patient. The FDG avidity score was three (i.e., uptake slightly or moderately higher than liver uptake) in one biopsied lesion (1.1%) and four (i.e., uptake markedly higher than liver uptake) in 87 biopsied lesions (98.9%).

### 3.3. Biopsy

Fifty-one lesions (58.0%) were sampled under CT guidance, and 37 lesions (42.0%) were sampled under US guidance. Biopsied lesions were located in the lung (*n* = 41), liver (*n* = 17), neck lymph nodes (*n* = 14), bone (*n* = 11), muscle (*n* = 2), abdominal wall (*n* = 1), mediastinal lymph nodes (*n* = 1), and mesentery (*n* = 1). All tissue samplings were histologic core needle biopsies, except for nine neck lymph node samplings that were done with cytologic fine needle aspirations. Seventy-eight tissue samplings (88.6%) were without any reported complications, whereas transient complications were reported to have occurred during ten tissue samplings (intrapulmonary bleeding with hemoptysis (*n* = 4) and pneumothorax (*n* = 6)).

### 3.4. Nature of Biopsied Lesions

**Figure 1:**
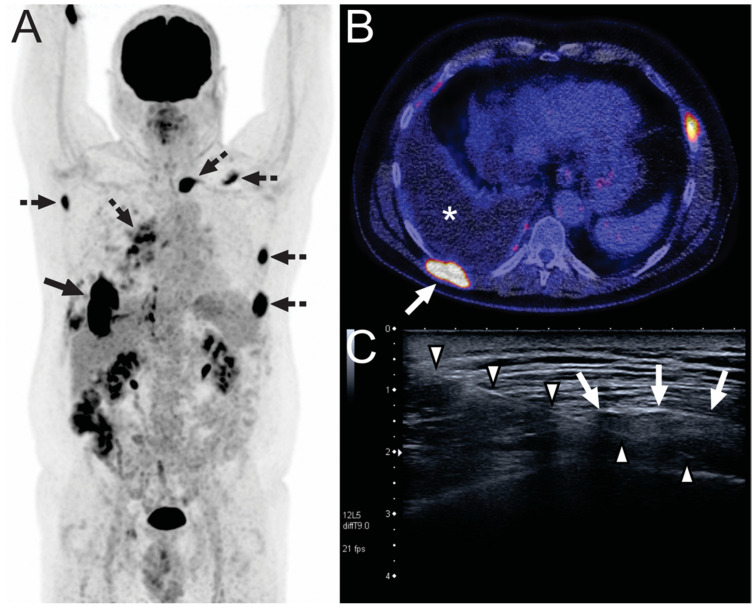
A 67-year-old man with stage IIIA right upper lobe lung adenocarcinoma was treated with chemotherapy and radiation therapy. Four months after completion of therapy, a CT scan (not shown) suggested tumor progression with a right-sided pleural fluid collection, for which a (talc) pleurodesis was performed. Subsequently, he was referred to our tertiary care center for immunotherapy with crizotinib. Another four months later, FDG-PET was performed for response evaluation. FDG-PET (**A**) showed multiple FDG-avid pleural and extrapleural lesions (dashed arrows), the largest at the right dorsal side of the chest wall (continuous arrow), suspicious for metastatic disease. The largest extrapleural lesion on the right dorsal side of the chest wall is also seen on fused FDG-PET/CT ((**B**), arrow), along with a pleural effusion (asterisk). Pleural fluid aspiration showed a lymphocytic exudate without any malignant cells. Subsequently, ultrasound-guided biopsy was ordered by the pulmonary oncologist for lung cancer mutation analysis. The ultrasound image (**C**) shows the biopsy needle (arrowheads) piercing the extrapleural lesion on the right dorsal side of the chest wall (arrows). Pathologic examination revealed a multinucleated giant cell reaction due to talc particles and no lung cancer.

Pathological analysis revealed a malignancy in 83 (94.3%) of 88 biopsies and one benign lesion (1.1%). The malignancies identified by pathological analysis were lung cancer (*n* = 78), melanoma (*n* = 1), gastrointestinal stromal tumor (*n* = 1), diffuse large B-cell lymphoma (*n* = 1), colon cancer (*n* = 1), and breast cancer (*n* = 1). The single benign lesion was a foreign body giant cell granuloma secondary to talc exposure (Figure 1). Four of 88 biopsies were considered inconclusive, but biopsy was not repeated. Based on clinical and imaging follow-up, three of these lesions could be classified as malignant; these cases concerned metastases from lung cancer (*n* = 2) and primary lung cancer (*n* = 1). For one biopsy, the follow-up could not provide sufficient confirmation for a diagnosis. This result was excluded from analysis.

**Figure 2:**
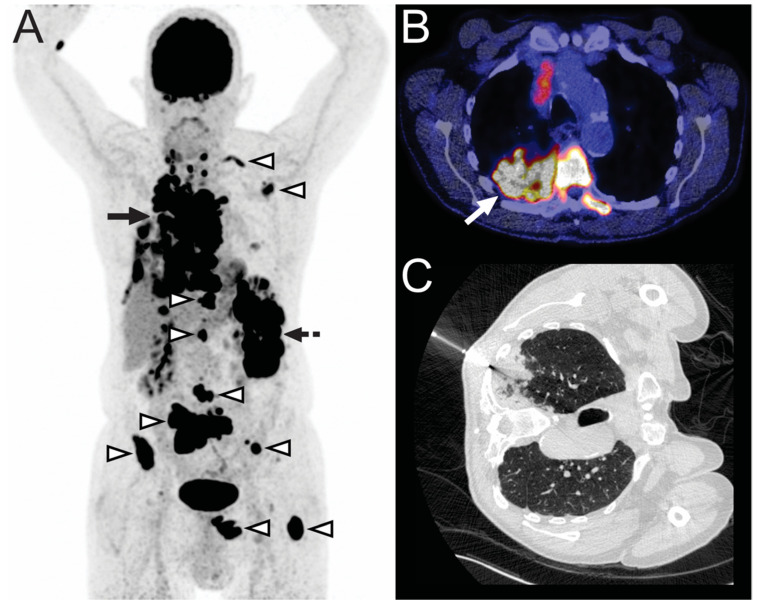
A 78-year-old man with a lesion in the right upper lobe, suspicious for lung cancer, was treated with radiation therapy, although no pathologic diagnosis was made. Two months later, a CT scan (not shown) suggested tumor progression. Subsequently, he was referred to our tertiary care center. FDG-PET (**A**) showed an FDG-avid mass in the right lung (continuous arrow), FDG-avid lesions in the spleen (dashed arrow), and multiple FDG-avid bone lesions (arrowheads), suspicious for metastatic disease. The mass in the right lung is also shown on fused FDG-PET/CT ((**B**), arrow). CT-guided biopsy was ordered by the pulmonary oncologist for lung cancer mutation analysis. CT image (**C**) shows the biopsy needle just before tissue sampling of the mass in the right lung. Pathologic examination revealed diffuse large B-cell lymphoma and no lung cancer.

**Figure 3:**
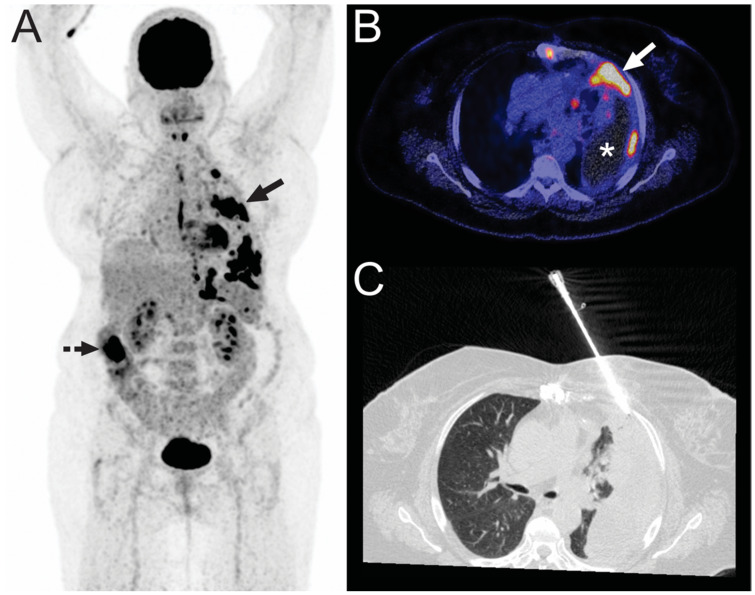
A 70-year-old woman with a known left-sided pleural effusion and a suspicion of lung cancer based on previous chest radiography and CT (not shown) was referred for FDG-PET. FDG-PET (**A**) showed multiple pleural lesions (continuous arrow), also demonstrated at fused FDG-PET/CT ((**B**), arrow), along with a pleural effusion ((**B**), asterisk). FDG-PET also showed an FDG-avid area in the ascending colon, which was interpreted as either physiological or due to diverticulitis or colitis by the attending nuclear medicine physician. CT-guided biopsy was ordered by the pulmonary oncologist to confirm the diagnosis of lung cancer and mutation analysis. CT image (**C**) shows the biopsy needle just before tissue sampling of one of the pleural lesions. Pathologic examination revealed intestinal adenocarcinoma and no lung cancer. Colonoscopy was performed, which demonstrated a tumor in the ascending colon that proved to be a well- to moderately-differentiated adenocarcinoma after biopsy.

Interestingly, in two cases, an unsuspected malignancy was diagnosed. One case involved a 77-year-old male who presented with a pulmonary lesion as detected on CT, which was thought to represent lung cancer even after FDG-PET/CT, but proved to be diffuse large B-cell lymphoma upon biopsy (Figure 2). The other case concerned a 69-year-old female who presented with pulmonary lesions as seen on chest radiography, which was thought to represent lung cancer even after FDG-PET/CT, but proved to be metastatic colonic adenocarcinoma upon biopsy (Figure 3).

### 3.5. PPV of FDG-PET/CT for Malignancy

The PPV of FDG-PET/CT for malignancy in biopsied FDG-avid lesions was 98.9% (95% CI: 93.8–99.8%). For patients with an oncological history, the PPV was 98.7% (95% CI: 92.9–99.8%), and for patients with no oncological history, the PPV was 100% (95% CI: 74.1–100.0%). There was no significant difference between the PPV of the group with an oncological history and the group without an oncological history (*p* = 0.71).

## 4. Discussion

The results of this study indicate that FDG-PET/CT achieves a very high PPV for malignancy (98.9%) when the clinical suspicion of malignancy is high. No significant difference was observed between the PPV in patients with no oncological history (100%) and the PPV in patients with an oncological history (98.7%) (*p* = 0.71). These results may question the need to perform invasive tissue sampling to confirm malignancy when FDG-avid disease is encountered in a patient with a clinical suspicion of cancer. However, caution is warranted, because the PPV for malignancy did not reach 100%, since one case unexpectedly proved to be due to a foreign body giant cell granuloma secondary to talc exposure. In addition, there were two cases in this series of 88 included biopsies where an unexpected other malignancy was diagnosed (T-cell histiocyte rich large B-cell lymphoma and metastatic colon adenocarcinoma, which both have a different prognosis and require a different treatment than lung cancer, which was the initial presumed diagnosis). Therefore, we postulate that tissue sampling remains an essential part of the diagnostic work-up of patients with FDG-avid disease, regardless of the clinical suspicion of (a certain) malignancy, and that no management decisions should be made without pathological confirmation. In addition, patients who are referred for biopsy and mutation analyses should be informed that unexpected benign pathology or an alternative malignant diagnosis may emerge after the acquired tissue has been pathologically analyzed. Emphasis on this part of patient counselling is important as anxiety is highly prevalent in patients waiting for results of biopsy, reinforcing the need for adequate patient information [13,14].

A previous study by Gupta et al. [10] investigated 195 biopsied FDG-avid lesions in 193 patients with a suspected or proven malignancy. Gupta et al. [10] reported FDG-avid lesions to be malignant in 82.4% of cases. Another study by Nguyen et al. [9] included 227 patients with 231 biopsied extrapulmonary FDG-avid lesions and found an overall PPV of FDG-PET/CT for malignancy of 72%. Lesion location significantly affected the PPV (*p* < 0.001); bone (96%) and liver (90%) lesions had significantly higher PPVs for malignancy compared with other locations, whereas lymph nodes (60%) had a significantly lower PPV for malignancy [9]. In addition, lesions that were morphologically suspicious and morphologically benign according to CT findings alone were associated with PPVs of 74% and 57%, respectively (*p* = 0.05) [9]. Yet another study by Harders et al. [15] included 168 patients with a solitary pulmonary lesion suspicious for lung cancer on CT. Before biopsy, all patients underwent FDG-PET/CT, which reportedly yielded a PPV for malignancy of 89% [15]. Compared to these previous studies [9,10,15], the PPV found in the present study is higher (98.9% compared to 82.4% [10], 71.9% [9], and 89% [15]). This difference can be explained by differences in the study populations, given the fact that the present study only included patients with a high clinical suspicion for malignancy as defined by the requested mutation analysis, which might have resulted in a higher incidence of malignancy. Furthermore, in the present study, the two main locations for biopsy were the lung (46.6%) and liver (19.3%), where FDG-avid lesions have been shown to be more likely malignant than in other locations such as lymph nodes [9,15].

This study had some limitations. First, the majority of included patients were evaluated because of a suspicion of lung cancer, which may decrease the generalizability of the PPV that was found for other patient categories. Second, this study was performed in a tertiary care university center, where the probability of finding a malignancy in patients referred for FDG-PET/CT scanning may be higher than in non-academic hospitals, which may also have affected the results. Therefore, further research may be necessary to make more accurate recommendations on the need for biopsy as the gold standard for the confirmation of malignant FDG-avid disease in different patient populations.

In conclusion, although the PPV of FDG-PET/CT for malignancy in patients with a clinical suspicion of active malignant disease is very high, biopsy is still recommended to avoid inappropriate patient management due the non-negligible chance of dealing with FDG-avid benign disease or unexpected other malignancies.

## Figures and Tables

**Table 1:** Indication for FDG-PET/CT scanning per patient category.

Indication for Current FDG-PET/CT Request	History of Malignant Disease	Number	Percentage
Suspected lung cancer	None	11	13.3%
Suspected new or progressive lung cancer	Lung cancer	56	67.5%
	Lung and esophageal cancer	1	1.2%
	Lung and prostate cancer	1	1.2%
	Lung cancer and lymphoma	2	2.4%
	Lung and breast cancer	1	1.2%
	Lung and laryngeal cancer	1	1.2%
	Lung and squamous cell carcinoma	1	1.2%
	Lung, prostate, and rectum cancer	1	1.2%
	Sarcoma and skin squamous cell carcinoma nose	1	1.2%
	Breast and ovarian cancer	1	1.2%
	Colon cancer	1	1.2%
Suspected new or progressive melanoma	Melanoma	1	1.2%
Suspected new or progressed gastrointestinal stromal tumor	Gastrointestinal stromal tumor	1	1.2%
Suspected new or progressive esophageal cancer	Lung and esophageal cancer	1	1.2%
Pathological lymph nodes	Melanoma	1	1.2%
Unknown primary tumor	Cancer of unknown primary	1	1.2%

## Data Availability

The data presented in this study are available upon request from the corresponding author. The data are not publicly available due to privacy restrictions.

## Abbreviations

PET	positron emission tomography
CT	computed tomography
FDG	^18^F-fluoro-2-deoxy-D-glucose
PPV	positive predictive value
US	ultrasonography
CI	confidence interval
